# A Genetic Variant in the Distal Enhancer Region of the Human Renin Gene Affects Renin Expression

**DOI:** 10.1371/journal.pone.0137469

**Published:** 2015-09-14

**Authors:** Yasukazu Makino, Tadashi Konoshita, Atsuhito Omori, Nobuhiro Maegawa, Takahiro Nakaya, Mai Ichikawa, Katsushi Yamamoto, Shigeyuki Wakahara, Tamotsu Ishizuka, Tamehito Onoe, Hiroyuki Nakamura, Genomic Disease Outcome Consortium (G-DOC) Study Investigators

**Affiliations:** 1 Third Department of Internal Medicine, University of Fukui, Faculty of Medical Sciences, Fukui, Japan; 2 Division of Rheumatology, Department of Internal Medicine, Kanazawa University, Graduate School of Medical Science, Kanazawa, Japan; 3 Department of Environmental and Preventive Medicine, Kanazawa University, Graduate School of Medical Science, Kanazawa, Japan; University of Louisville, UNITED STATES

## Abstract

**Background:**

The high heritability of plasma renin activity was confirmed in recent investigations. A variation located near the strong enhancer of the human renin gene (*REN*), C-5312T, has been shown to have different transcription activity levels depending on its allele: the 5312T allele shows transcription levels that are 45% greater than those of the 5312C allele. The purpose of this study was to confirm the hypothesis that variations in the enhancer region of the *REN* gene are involved in regulating renal expression of renin.

**Methods:**

Sixty-four subjects with biopsy-proven renal diseases were included in this study (male/female: 35/29, age 41.9 ± 20.9 years, SBP/DBP 123.1 ± 23.7/73.4 ± 14.8 mmHg, s-Cr 0.93 ± 0.63 mg/dl). A genetic variant of *REN*, C-5312T, was assayed by PCR-RFLP and the TaqMan method. Total RNAs from a small part of the renal cortex were reverse-transcribed and amplified for *REN* and *GAPDH* with a real-time PCR system.

**Results:**

Logarithmically transformed expression values of the relative ratio of *REN* to *GAPDH* (10^−3^) were as follows (mean ± SE): CC (26 cases), 0.016 ± 0.005; CT (33 cases), 0.047 ± 0.021 (p = 0.41 vs. CC); TT (5 cases), 0.198 ± 0.194 (p = 0.011 vs. CC, p < 0.031 vs. CT). Thus, significant differences in *REN* expression were observed among the genetic variants.

**Conclusion:**

The results suggest that variants in the enhancer region of the human renin gene have an effect on the expression levels of renin in renal tissue; this observation is in good accordance with the results of the transcriptional assay.

## Introduction

The renin-angiotensin system (RAS) plays pivotal roles in blood pressure regulation,[1] cardiovascular, renal and metabolic conditions[2–4] and pharmacological status.[5, 6] One of the major rate-limiting steps of this system is the conversion of angiotensinogen to angiotensin I, which is catalyzed by renin. Thus, the regulation of renin gene expression is thought to be fundamental to the regulation of the total system. Recent studies also confirmed that a higher plasma renin level was associated with greater cardiovascular mortality in patients referred to coronary angiography[7] and in community-based cohort studies.[8] High heritability estimates were reported for plasma renin activity in an investigation.[9] These observations are presumed to be attributed to genetic variants, especially those in the promoter locus that contributes to transcriptional activity. Previously, six protein-binding sites had been mapped and characterized in the proximal promoter region of the human renin gene (*REN*) (-336 to +16) by DNase I footprint assay with nuclear extracts from human chorionic cells or ischemic human renal cortex.[10–14] These evaluations revealed the physiological mechanisms of the *cis*-elements; however, there have been no studies on genetic variants in the proximal promotor region. On the other hand, a strong enhancer in the human *REN* promoter region, located 5777–5552 nucleotides upstream from the transcription start site, has been described.[15] A variant located near this enhancer, C-5312T, has been shown to have a different transcription activity level according to its alleles in choriodecidual cells, a model of renin-producing cells. The levels of transcription were 45% greater with the 5312T variant than with the 5312C variant.[16] Recently, we have demonstrated that variations in the renin gene were associated with plasma renin activity as a genetic factor[17] and also that such variations are pharmacogenetic determinants of the antihypertensive effect of angiotensin receptor blockers.[18, 19]

The purpose of this study was to confirm the hypothesis that this validated enhancer region variant is associated with renal *REN* expression. Thus, genetic variants and the renal *REN* mRNA from renal biopsy specimens were assayed.

## Methods

### Subjects and treatments

This study was undertaken in accordance with the Declaration of Helsinki Principles. The study protocol was approved by the ethics committee of the University of Fukui (No. 17–12, 13–1 and 14–2). Written informed consent to participate in this study was provided by all participants. Subjects were 64 patients with biopsy-proven renal conditions, including 6 with benign nephrosclerosis, 7 with minor lesions, 35 with primary glomerulonephritis, 10 with diabetic lesions, 5 lupus erythematodes and 1 with interstitial nephritis.

For each subject, salt intake was standardized to 6 to 10 g daily during hospitalization according to the hypertension status. The numbers of patients administered with anti-hypertensive agents were as follows: calcium channel blocker (CCB), 8; alpha-blocker (alpha B), 7; diuretics, 5; ACE inhibitor (ACEI), 1; angiotensin receptor blocker (ARB), 1. However, ACEI and ARB were replaced by CCB or alpha B at least 1 week prior to biopsy.

### Genetic variant analysis

Genomic DNA was isolated by phenol-chloroform extraction or with a QIAamp DNA Blood Mini Kit (QIAGEN Inc., Tokyo, Japan) from whole blood drawn from subjects with EDTA-2Na. In DNA amplification procedures for the genetic variant assay, a thermal cycler (ASTEC, PC-700, Fukuoka, Japan) was used.

For early samples, the *REN*, C-5312T, was assayed by PCR-RFLP with *Dde*I digestion. The primers used were as follow: sense oligonucleotide, 5’-CGTAGTGCCATTTTTAGGAAC-3’, and anti-sense oligonucleotide, 5’-AACACCAAAGCAGGCTTAA-3’. The PCR program consisted of 30 cycles of denaturation at 94°C for 30 seconds, annealing at 55°C for 30 seconds and extension at 72°C for 30 seconds followed by a final extension at 72°C for 5 minutes. Samples were then incubated with *Dde*I overnight at 37°C. To examine the sizes of the PCR products, after the addition of loading buffer containing 10% glycerol, 10 μl of the mixture was loaded onto agarose gels.

**Fig 1:**
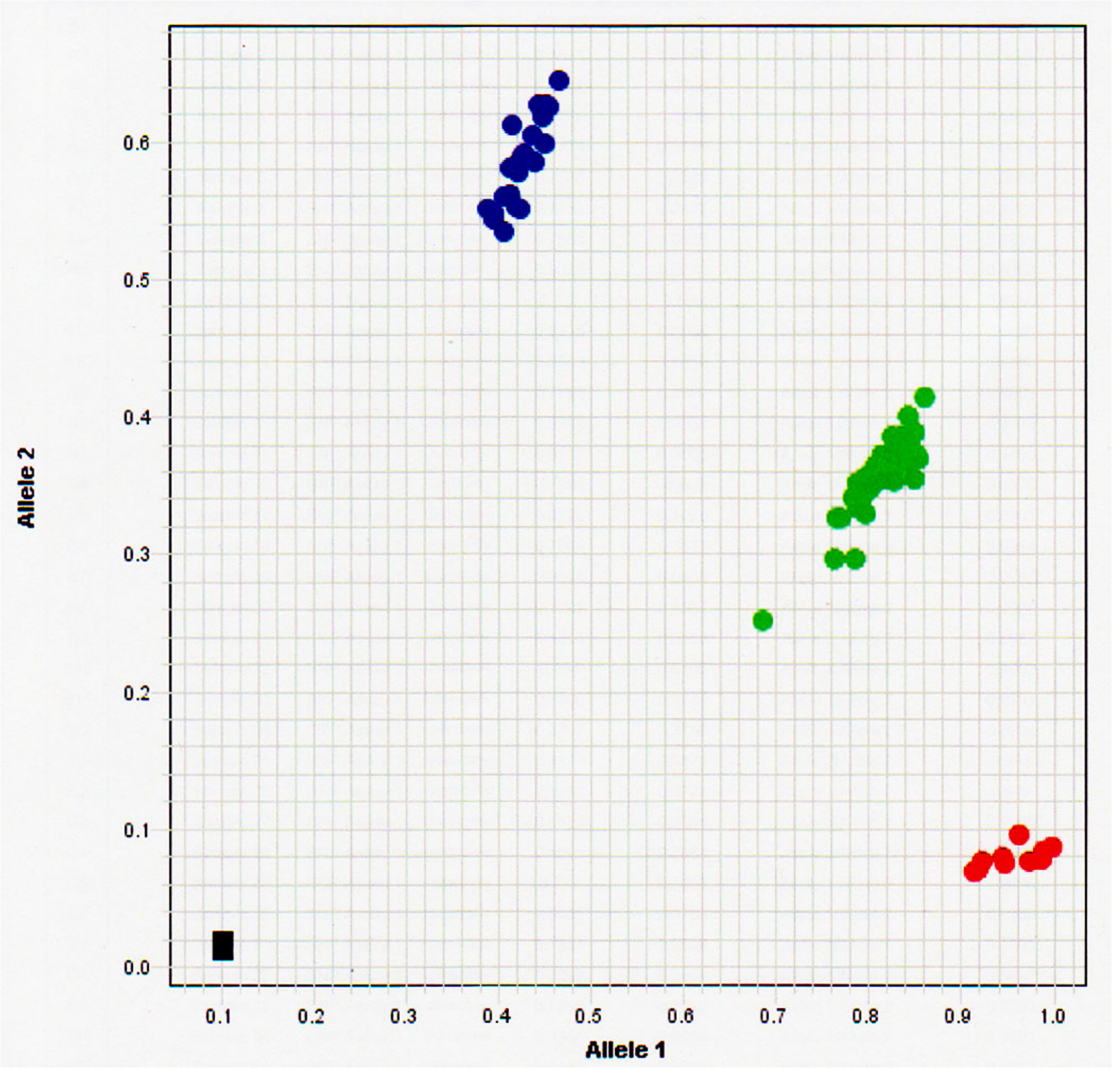
Assay of renin C-5312T determined by the TaqMan system. Allele 1 indicates the T allele and allele 2 indicates the C allele. Blue, green, and red dots indicate CC, CT, and TT genotypes, respectively. The two black squares represent the negative control for which water was used instead of DNA samples.

For recent samples, the variant was assayed using the TaqMan method using the common PCR conditions recommended by the manufacturer (Applied Biosystems, Foster City, CA, USA) with a custom-made primer set. The primers used were as follow: sense oligonucleotide, “ACTAGGAATCCAGGAGAATAGGTCTTT” and anti-sense oligonucleotide, “CCTTAGAACACCAAAGCAGGCTTA”. (Fig 1).

### Expression assay by real-time quantitative RT-PCR

**Fig 2:**
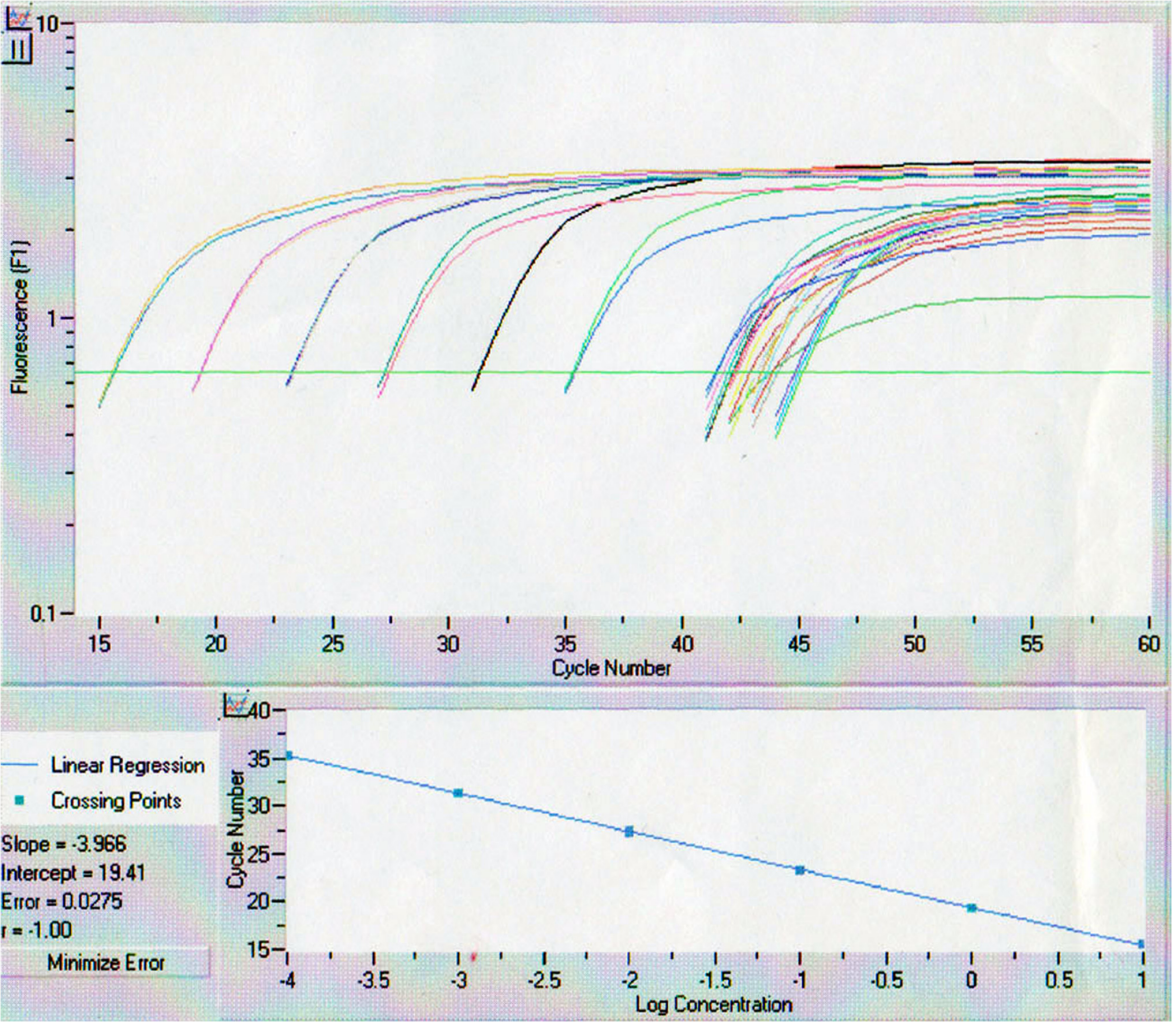
Measurement of renin mRNA by real-time PCR using a LightCycler system. Six orders of linearity were obtained.

Renal RNAs were extracted from a very small sample (approximately 2 mm of an 18G needle specimen) of renal cortex tissue obtained by percutaneous renal biopsy under echography. Almost all of these specimens contained approximately 20 to 30 glomeruli. After collecting the biopsy specimens, total RNAs were immediately extracted by using a commercial extraction kit, RNA-Bee (TEL-TEST, Inc., Friendswood, USA), according to the manufacturer’s protocol. The cDNAs were synthesized by reverse transcription with 500 ng/μl of Oligo-dT (TOYOBO Inc., Osaka, Japan) and reverse transcriptase (M-MLV) (TOYOBO Inc., Osaka, Japan). The synthesized cDNAs were amplified for renin as the target gene and GAPDH as a house-keeping gene. The primer sequences were: renin, 5’-GTGTCTGTGGGGTCATCCACCTTG-3’ (sense) and 5’-GGATTCCTGAAATACATAGTCCGT-3’ (anti-sense); GAPDH, 5’-CCCATCACCATCTTCCAGGAG-3’ (sense) and 5’-GTTGTCATGGATGACCTTGGC-3’ (anti-sense). The real-time PCR reactions took place in a reaction volume of 20 μl containing 0.5 mM of each primer and 2 μl of cDNA as a template in 2× QuantiTect PCR Master Mix (QIAGEN Inc., Tokyo, Japan). Measurements of the specific mRNAs of renin and GAPDH were performed by using a LightCycler System (Roche Diagnostics Inc., Tokyo, Japan). Each sample was measured in duplicate. Absolute quantification was performed using prepared concentration-known control samples (Fig 2). The mRNA levels were expressed as relative values to GAPDH mRNA and then logarithmically transformed.

### Statistical analyses

**Fig 3:**
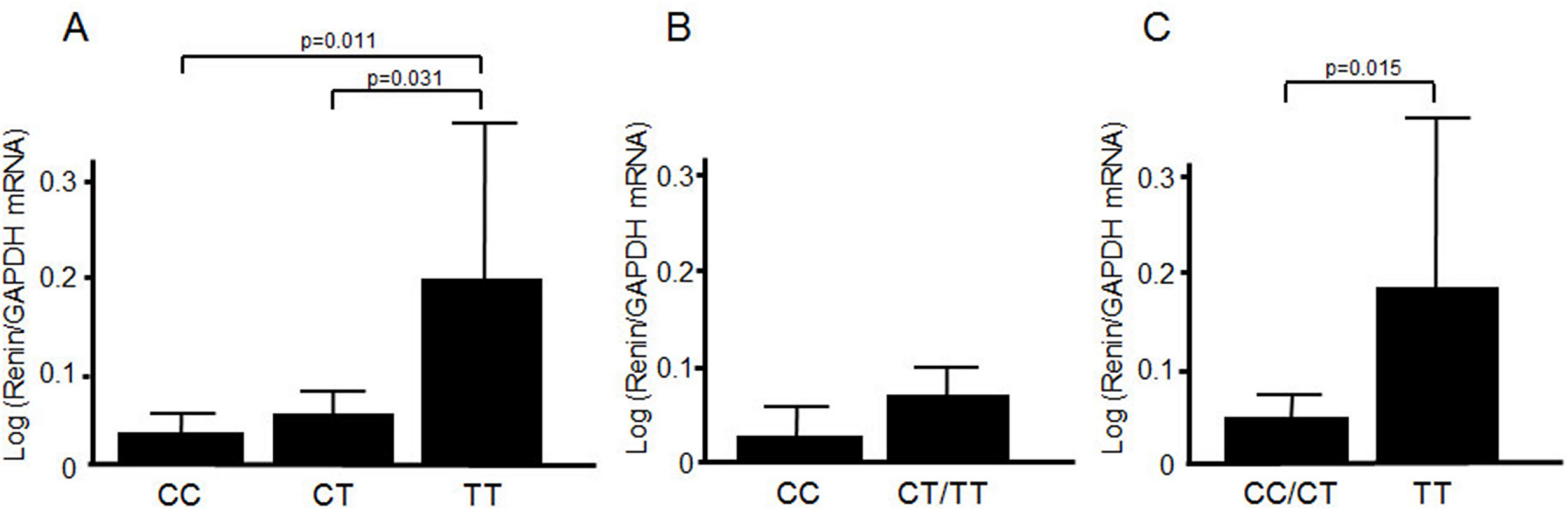
Genetic variants of *REN* and their gene expressions in renal tissue. mRNA for *REN* from human renal tissue was measured and compared among genotypes of C-5312T at the distal enhancer site. The values for genetic expression are expressed as logarithmically transformed values of their relative ratio to GAPDH (10^−3^). Columns and bars show the means and standard errors. The values were as follow (means ± SE): CC, 0.016 ± 0.005; CT, 0.047 ± 0.021 (p = 0.41 vs. CC); TT, 0.198 ± 0.194 (p = 0.011 vs. CC, p < 0.031 vs. CT) (A). The results of the comparisons between genotypes were as follows: CC and CT/TT (0.067 ± 0.031), p = 0.177 (B); CC/CT (0.033 ± 0.012) and TT, p = 0.015 (C).

Statistical analyses were performed with SPSS (ver. 17.0; SPSS Japan, Inc., Tokyo, Japan). Data are expressed as numbers, percentages, means ± SD, or medians (interquartile ranges), as appropriate. The differences between the variables of two or three groups were analyzed by ANOVA or Wilcoxon’s signed rank test, as follows: systolic blood pressure (SBP), diastolic blood pressure (DBP), serum creatinine level (s-Cr), eGFR, plasma renin activity (PRA), and plasma and aldosterone concentration (PAC) were analyzed by ANOVA; urinary sodium excretion, urinary potassium excretion, and urinary chloride excretion were analyzed by Wilcoxon’s signed rank test. Logarithmically transformed values of the relative expression of *REN* compared to that of *GAPDH* (10^−3^) for each genotype were evaluated by ANOVA (Fig 3). We performed comparisons between two genotypic groups as well as between three genotypic groups to better evaluate dominant and recessive models.

## Results

### Basic clinical characteristics of the subjects

The basic clinical characteristics of the subjects were as follows (Table 1) (values are means ± SD or means (interquartile), as appropriate): age, 41.9 ± 20.9 years; SBP, 123.1 ± 23.7 mmHg; DBP, 73.4 ± 14.8 mmHg; s-Cr, 0.93 ± 0.63 mg/dl; eGFR, 83.4 ± 35.3 ml/min/1.73 m^2^; PRA, 2.7 ± 1.3 ng/ml/h; PAC, 91.6 ± 49.7 pg/ml; urinary albumin excretion, 266.7 (44.2 − 682.8) mg/gCr; urinary sodium excretion, 105.3 (83.5 − 157.9) mEq/gCr; and urinary potassium excretion, 30.9 (25.6–38.5) mEq/gCr. Other major clinical characteristics are also listed in Table 1.

**Table 1:** Subjects characteristics.*

Characteristics	
Number	64
Female gender—no. (%)	29 (45.3%)
Age—yr	41.9±20.9
Body-mass index†	23.5±3.9
Blood pressure, mmHg	
Systolic	123.1±23.7
Diastolic	73.4±14.8
Serum sodium—mEq/l	139.9±2.8
Serum potassium—mEq/l	4.1±0.3
Serum chloride—mEq/l	103.4±3.0
Serum creatinine—mg/dl	0.93±0.63
eGFR—ml/min/1.73m^2^	83.4±35.3
Plasma renin activity, ng/ml/hr	1.7±1.3
Plasma aldosterone concentration, pg/ml	91.6±49.7
Urinary albumin excretion—mg/creatinine‡	266.7 (44.2–682.8)
Urinary sodium excretion—mEq/creatinine‡	105.3 (83.5–157.9)
Urinary potassium excretion—mEq/ creatinine‡	30.9 (25.6–38.5)
Urinary chloride excretion—mEq/ creatinine‡	105.8 (77.7–148.1)

*Plus-minus values are means ± SD.

†The body-mass index is the weight in kilograms divided by square of the height in meters.

‡Values shown are medians (interquartile ranges).

eGFR: estimated glomerular filtration rate.

### Genetic variants and clinical characteristics

The prevalence of the C-5312T genotypes were as follows: CC, 26 cases; CT, 33 cases; TT, 5 cases. The distributions were similar to those predicted from the Hardy Weinberg equilibrium. The main characteristics of each genotype are shown in Table 2. No significant difference was observed in these clinical characteristics among genotypes.

**Table 2:** Genetic variations in *C-5312T* and clinical characteristics.*

Characteristics	CC	CT	TT
Number	26	33	5
Blood pressure, mmHg			
Systolic	123.0±23.1	123.8±24.7	119.0±23.8
Diastolic	74.5±13.9	72.9±16.0	71.2±14.3
Serum creatinine—mg/dl	0.85±0.41	1.01±0.79	0.75±0.10
eGFR—ml/min/1.73m^2^	87.4±37.2	80.0±34.9	85.8±31.2
Plasma renin activity, ng/ml/hr	1.8±1.6	1.6±1.2	1.9±0.7
Plasma aldosterone concentration, pg/ml	90.4±48.7	94.1±54.2	81.2±19.9
Urinary sodium excretion—mEq/creatinine†	106.3 (99.1–159.2)	101.9 (80.3–154.4)	107.3 (94.4–128.8)
Urinary potassium excretion—mEq/ creatinine†	32.9 (27.3–44.1)	29.6 (24.0–36.5)	35.6 (32.1–60.8)
Urinary chloride excretion—mEq/ creatinine†	114.1 (89.5–162.9)	105.3 (74.7–145.3)	104.7 (101.2–149.3)

*Plus-minus values are means ± SD.

†Values shown are medians (interquartile ranges).

eGFR: estimated glomerular filtration rate.

### Genetic variants and the gene expression in renal tissue of *REN*

*REN* expression was measured at 10^−3^ order that of *GAPDH* expression. The values were as follow (means ± SE): CC, 0.016 ± 0.005; CT, 0.047 ± 0.021 (p = 0.41 vs. CC); and TT, 0.198 ± 0.194 (p = 0.011 vs. CC, p < 0.031 vs. CT) (Fig 3). For the comparison between C allele homozygotes and CT/TT, the values were 0.016 ± 0.005 and 0.067 ± 0.031, respectively (p = 0.177). For the comparison between CC/CT and T allele homozygotes, the values were 0.033 ± 0.012 and 0.198 ± 0.194, respectively (p = 0.015). After Bonferroni’s correction for the examination of three genotypic groups (CC, CT, and TT), the difference between CC and TT was significant. Thus, a significant difference in *REN* expression in renal tissue was observed between genetic variants of C-5312T.

## Discussion

Recently, genetic variants have been studied for association with their phenotypic characteristics in a large number of physiological and pathological conditions. In particular, variants of the components of RAS have been evaluated in cardiovascular and renal disorders.[20–26] These early emerging variants have been validated mainly by evaluating plasma concentrations as intermediate phenotypes.[21, 27] However, only a small number of recent reports have evaluated the validness of variants. The elucidation of the tissue expression levels of targeted genes is thought to be informative for such validation of genetic variants. Renin gene expression in human renal tissues has been evaluated quantitatively,[28] but no such study has been conducted with genetic variants. *REN* C-5312T is one of the rare validated genetic variants with reports that have shown their effects at the genetic transcriptional level[16] and also at the plasma activity level.[17] In this study, we evaluated the renal expression level of *REN* in human renal tissues in respect to *REN* genetic variants for the first time. Tissue expression levels of *REN* were significantly higher in T allele homozygotes than in heterozygotes and C allele homozygotes. This observation is in good accordance with the results of the transcriptional assay. Thus, C-5312T can now be regarded as validated in tissue expression level as well as in transcriptional level and plasma activity level.

Renin is thought to be excreted via a regulated pathway and its excretion and synthesis is largely affected by physiological status, especially the posture.[29] At renal biopsy, our subjects were prone for at least 30 minutes before the renal specimens were taken so that our measurements of mRNA would represent the expression levels of the basal status. Consequently, C-5312T is thought to be related to the expression of *REN* at the basal status. It is possible that physiologically exaggerated status, for example, standing, alters the relationship between the genotype and gene expression.

High heritability estimates were reported for plasma renin activity in a recent investigation,[9] and we have also demonstrated in a recent report that a renin gene variant was associated with plasma renin activity as a genetic factor [17]. Each PRA value by genotype for C-5312T tended to be in fair accordance with the tissue expression levels; however, they did not reach the level of statistical significance in this study. Associations between genetic variants of *REN* and hypertension were not recognized in early studies,[30–32], but in recent studies, they have been recognized for several conditions including hypertension,[33, 34] primary aldosteronism [35] and diabetic nephropathy.[36]

Several limitations of the present study should be described. Although the tissue renin expression levels were log-transformed, the distribution was still not normal. The sample number in this study was relatively small, so differences in disease states could not be assessed by genotype. Cessation of ACEI and ARB for 1 week may have been sufficient to normalize renin expression. Although a sample population comprising individuals with various genetic backgrounds would have been ideal, this study comprised only Japanese subjects. One other limitation was that we investigated only one variant of *REN*.

In conclusion, the results suggest that variants in the enhancer region of the human renin gene have an effect on the expression levels of renin in renal tissue; this observation is in good accordance with the results of the transcriptional assay. Thus, the validated C-5312T of *REN* may be a candidate gene for susceptibility to cardiovascular, renal and metabolic conditions. Closer examination in a larger population is necessary to solve these issues.

## Supporting Information

S1 File All raw data of the subjects. (XLSX)
